# Minding menopause in patients with cognitive impairment: a patient’s perspective and reflections on clinical practice

**DOI:** 10.1186/s12905-023-02770-w

**Published:** 2023-11-10

**Authors:** Elias Thomas, Petra Verdonk, Hanneke Rhodius-Meester, Majon Muller

**Affiliations:** 1 Department of Internal Medicine, Geriatrics Section, Amsterdam Cardiovascular Science, Amsterdam University Medical Centre, Amsterdam UMC, De Boelelaan 1117, 1081HV, Amsterdam, the Netherlands; 2 Amsterdam Public Health Institute, Ageing & Later Life, Amsterdam UMC, Amsterdam, the Netherlands; 3 Department of Ethics, Law and Humanities, Amsterdam UMC, VU University Amsterdam, Amsterdam Public Health Research Institute, Amsterdam, the Netherlands; 4 Department of Neurology, Amsterdam Neuroscience, Alzheimer Center Amsterdam, VU University Amsterdam, Amsterdam UMC, Amsterdam, the Netherlands; 5 Department of Geriatric Medicine, The Memory Clinic, Oslo University Hospital, Oslo, Norway; 6 Amsterdam Cardiovascular Sciences, Atherosclerosis & Ischemic Syndromes, Amsterdam, the Netherlands

**Keywords:** Menopause, Women’s health, Patient narratives, Cognitive complaints, Health inequalities, Stigma, Clinical practice

## Abstract

Many women experience impairment in cognitive function during perimenopause. These symptoms are often not attributed to perimenopause by women themselves, by family and friends, or by healthcare professionals. In this article, we present a case in which perimenopausal complaints were attributed to early dementia and discuss mechanisms contributing to the low level of awareness of perimenopausal symptoms amongst patients and clinicians. Stigma amongst women and healthcare professionals impairs the recognition of perimenopausal complaints, and consideration of treatment options by clinicians. We advocate raising awareness in patients and physicians with more education, and the incorporation of potential menopause-related symptoms in general clinical guidelines.

## Introduction

Menopause represents a challenging time for many: fluctuations in estrogen levels may lead to symptoms such as hot flashes, night sweats, and vaginal dryness [1]. Perimenopause is also associated with changes in cognitive function, including impairment of the ability to focus and to remember daily tasks or conversations [2]. We present a clinical case of cognitive impairment in which menopausal changes might be considered. The patient in question gave consent for the publication of her case description and fragments of the in-depth interview. We explore how and why possible menopause-related cognitive complaints may be overlooked or misclassified, and suggest ways in which patients and physicians may become more aware of the possibility that menopausal symptoms have an effect on cognitive functioning, and how. We conducted an in-depth interview with the patient concerned and we also reflect on current clinical practice.

### Case

M., a 56-year-old National Defense employee, was referred to our memory clinic, a tertiary care center, for a second opinion. Her medical history included a minor stroke ten years previously, after which she had not experienced any residual symptoms. She came to our clinic because she had been suffering from increasing lapses in memory for approximately five years. She was also having trouble focusing, difficulty with performing multiple tasks and difficulty following and remembering conversations with close family. At the time of the appointment, she was unable to work because of these symptoms and she needed help to perform routine tasks at home such as cleaning and cooking. Neuropsychological evaluation showed impaired processing speed and impaired memory performance, and her brain MRI showed diffuse vascular damage with confluent white matter hyperintensities (Fazekas grade 2, global cortical atrophy grade 1, medial temporal lobe atrophy grade 1 bilaterally, and no microbleeds). M. was diagnosed with early-stage vascular dementia, and appropriate supporting care was initiated [3]. However, shortly after visiting our clinic, she initiated hormone replacement therapy (HRT) after her family had read about the menopause on the internet. Within six months after starting HRT, she noticed a major improvement in her symptoms: *“After six months, I was a completely different person! […] I could function independently again […]. I could actually just do everything myself.”* Clinical evaluation and Mini Mental State Examination (MMSE) confirmed her subjective improvement, slightly rising from 27 to 29 points. Due to her clinical improvement, she did not meet the criteria for vascular dementia, and her diagnosis was reversed. At present – a year later – M. has no cognitive complaints, she performs her tasks of daily living independently, and she has returned to work.

### Menopause and cognitive function

Menopause is marked by fluctuations and an eventual drop in estrogen levels and is retrospectively determined after twelve months of amenorrhea. The period preceding menopause, in which changes in the menstrual cycle start to occur, is known as *perimenopause* and it normally lasts four to ten years [1]. Approximately two thirds of perimenopausal women^1^ experience cognitive symptoms such as memory problems, and attention or language deficits, alongside the more widely known vasomotor symptoms (VMS) such as hot flashes [1, 4]. There are many subjective reports of memory loss, and recent evidence has also shown objective cognitive impairment during perimenopause, especially in the domains of memory and attention [2, 5, 6]. In addition, perimenopause is associated with symptoms of mood disorders, such as depression, which in itself may lead to cognitive complaints, and it is closely associated with memory and attention deficits [7]. HRT could help to relieve perimenopausal symptoms, and to improve mood and sleeping patterns and thereby cognitive function [8]. The indications and precautions relating to HRT are outside the scope of this article and are clearly stated in various international guidelines [9–11]. Finally, studies have shown that education about the effects of the perimenopause on cognition significantly improves quality of life during perimenopause [12].

### Patients and stigmatization

Although roughly half of the people in the world go through menopause, research shows that this is still a stigmatized subject. Not only sexist, but also ageist, notions may constitute barriers to perimenopausal symptoms becoming visible in the public and medical domains. Health care is generally constructed around the perspective of non-elderly males. Women – and their bodies – are expected to fit into this male-oriented system, which is often seen as being as asexual or neutral. Female physiology, including menstruation, pregnancy, and menopause, is thought of as a complicating factor in health care and research, and is often set apart as belonging to a specific specialism such as gynecology. In clinical practice, however, all specialisms work with female patients and many specialisms require a basic knowledge of the effects of menopause to provide good care. The compartmentalization of menopausal complaints, and the corresponding lack of emphasis on these complaints in general medicine, may have adverse effects on patients when they present with symptoms which are not overtly linked to female physiology or anatomy – such as the cognitive impairment in M.’s story.

Cultural attitudes toward female-specific complaints may also play a role in the relative invisibility of perimenopausal symptoms. As with other attributes viewed as “women’s issues” such as menstruation, women who talk about menopausal symptoms are sometimes dismissed as behaving in exaggerated or even hysterical ways, and research has shown they may be perceived as less intelligent when mentioning these issues [13]. M. reported that she felt her complaints had not been taken seriously by her treating physician, in line with reports of stigmatization amongst healthcare professionals when considering menopause-related complaints [14].

*Well look, if you talk to a female doctor, they can sometimes relate, or may have educated themselves. The male doctors have an attitude of, well, they think it’s really all bit of a fuss about nothing. Really far-fetched.*

Women may internalize this cultural attitude and report feeling ashamed about their symptoms, with low self-esteem and impaired quality of life as a result.

The link between menopause and ageing may also lead to feelings of shame and anxiety. This negative connotation reflects cultural ideologies about ageing, classifying older women as less worthy, relevant or intelligent [15]. It is worthy of mention here that, in cultures where ageing is valued as a positive trait, women have a more positive attitude towards menopause, and even report less bothersome symptoms [16]. The combination of gender- and age-related stereotypes may represent an obstacle to the initiation of an open dialogue about menopausal symptoms in daily life and between patients and clinicians. Furthermore, because of the associated lack of awareness and knowledge, women may not attribute any symptoms to perimenopause. M.’s experience served to break open a dialogue of this kind in her social circles, revealing perimenopausal symptoms in friends and family that had not been discussed previously.

*Well, so that they did actually start to think about it a bit and they said, sort of, I’ve actually got […] those symptoms and I think I will […] go and talk to my doctor about them.*

### Healthcare professionals and stigmatization

Negative views about ageing are not only present – and persistent – in patients but also in physicians. Physicians often unconsciously assess older people and women as being less intelligent and less competent, and this is an impediment to an open patient-physician dialogue, possibly leading to ineffective communications about perimenopausal symptoms, and the incorrect interpretation of perimenopausal symptoms in women consulting their clinicians [17]. For example, because of a partial overlap in symptoms – fatigue, sleep problems, anxiety, mood disorder – a mistaken diagnosis of burn-out, or in this case even dementia, may be made, with the potential role of menopause not being considered. The presence of other social vulnerabilities may further exacerbate the risk of misdiagnosis. The concept of *multiple jeopardy* comes into play here: different social vulnerabilities found in conjunction, including female sex and gender, older age, language barriers and poor financial stability, cannot be viewed separately and, when combined, they may render patients especially susceptible to ineffective dialogue, misdiagnosis and adverse outcomes. Indeed, patients who deviate from the sociocultural normative image (e.g. middle-income or financially stable, white, cisgender and able-bodied), are less likely to have effective dialogue with their physician in which their complaints are heard and patient satisfaction is reached [18]. This may also be the case with perimenopausal symptoms. Studies show that menopause-related complaints are generally more severe in women with low education levels, women who live in rural settings or women with reduced financial stability [19]. To our knowledge, there are no studies looking at the prevalence of the misclassification of perimenopausal symptoms in groups with different sociocultural or socioeconomic backgrounds.

### Social impact

The personal impact in M.’s case is clear. However, the social impact of perimenopause is also evident in terms of women’s ability to work: over three-quarters of women report serious problems relating to the demands of their occupation [16]. Especially in women with severe perimenopausal symptoms, impaired employability is therefore a distinct possibility. For M., working in a male-centered environment such as National Defense may have had a further impact on her ability to discuss her symptoms and adapt her working environment to match her changing needs. Research shows that, in most work settings, adaptions of this kind are not addressed or considered normal. More often, women overcompensate – aiming to disprove the presumption that they might be less productive when experiencing “women’s issues” – and eventually reduce participation in paid work [20, 21]. This may further increase anxiety and impair quality of life as well as working performance.

### Education and awareness

Physicians play an important role in the normalization of the discussion of perimenopause and may complement it with professional subject-specific knowledge. Unfortunately, however, M.’s experience may well be more than an isolated case. Lack of awareness amongst clinicians impairs the recognition and acknowledgement of the symptoms experienced during perimenopause [22]. Although gynecologists are often better informed about the effects of menopause, other medical professionals are much less aware of perimenopausal symptoms and treatment options, and therefore less likely to recognize and treat them [23]. Awareness in the public domain could facilitate the discussion of perimenopausal symptoms in daily life and in clinical practice. It could also facilitate feelings of support and community for persons experiencing menopause, a factor that is associated with better quality of life [24]. M. is optimistic in this regard, having observed an increased awareness of perimenopause symptoms in younger generations.

*Look, with our generation […] you don’t talk about it of course […] you can just see that these are things that are talked about these days […] by younger people. Younger men and women.*

In addition to this heightened awareness in the public domain, clinicians could benefit from formal education about the menopausal transition. At present, doctors report that they have not been trained in this area and they feel badly equipped to deal with menopause in clinical practice [25]. Furthermore, although there are guidelines that address menopause separately, menopause is not mentioned in general clinical guidelines – once again assigning female physiology to a separate domain without the much-needed integration in the domain of general medicine, geriatrics or neurology [26]. Since menopause is also often not recorded or mentioned in patients’ clinical files, physicians receive little systematic encouragement to consider the role of menopause. In combination with the stigmatization of menopause that we have described among healthcare providers and patients, the potential role of menopause may therefore often be overlooked during the initial clinical assessment of patients.

### Implications for practice and policy

In conclusion, perimenopausal symptoms – especially in the cognitive domain – continue to be underrecognized in clinical practice and clinical education, and they may therefore be undertreated as well. In order to facilitate more openness about perimenopausal symptoms among patients and physicians, governments and healthcare authorities should facilitate public awareness campaigns about menopause, including subjects such as shame and societal stereotypes. A careful balance should be struck in the information provided between raising awareness about perimenopausal symptoms and possible alleviating treatment, and recognizing that menopause is a natural part of ageing. A qualitative study investigating the effects of listening to a menopause podcast series showed knowledge gain and a sense of community among participants, which led to increased self-confidence in discussing menopause-related symptoms with healthcare providers, as well as an increased acceptance of menopause, and even pride about their body’s “ability to grow and develop” [27]. Information about menopause should be attuned to a wide range of persons, including gender-diverse persons experiencing menopause and patients with low literacy levels. Furthermore, we recommend mandating the inclusion of menopause-related education in medical school curricula, as well as for general physicians, occupational health physicians, internists, geriatricians, and neurologists, leading to a better understanding and recognition among physicians of menopause-related cognitive symptoms, and more confidence about the treatment of these symptoms. More knowledge amongst healthcare providers will help to counter stereotypical thinking, facilitating better physician-patient dialogue in which the impact and treatment options during menopause may be discussed using a shared-decision approach. For M., structural awareness amongst physicians about the relationship between menopause and cognitive complaints could well have led to a much earlier improvement in her quality of life. The last decades, various international organizations, including the International Menopause Society (IMS) have developed resources and guidelines to further understanding of the impact of menopause and treatment options. In the Netherlands, the “H3-Network” – the 3 H’s signifying Head, Heart and Hormones – is a multidisciplinary network which connects healthcare professionals and experience experts to disseminate more knowledge on the effects of menopause and create more awareness amongst healthcare professionals and at a societal level [28]. For M., working in a male-oriented environment may also have contributed to the relative lack of focus on the role of menopause in her symptoms. Following earlier UK-based initiatives, the Dutch Menopause society recently released a guideline for employers on areas of concern and opportunities for improvement to facilitate adequate support for employees who are experiencing menopausal transition [29, 30]. In clinical care, awareness could be enhanced further by recording menopausal status in patient files. As far as we are aware, there are no initiatives which actively stimulate, facilitate or even mandate registration of menopausal status in patient files in the Netherlands or abroad. Breaking taboos and educating healthcare professionals about the menopausal transition and on how to discuss this with their patients will almost certainly lead to improvements in the care delivered. Furthermore, this information should be tailored to include women from a range of cultural, geographic and socioeconomic backgrounds so that they can obtain support and guidance during perimenopausal transition and embrace the next chapter in their lives with more confidence and a better quality of life.

## Data Availability

The datasets used and/or analyzed during the current study are available from the corresponding author on reasonable request.

## Footnotes

1 We use the word women in this article to refer to persons going through menopause, but non-binary persons, or other persons not identifying as a woman may also experience menopause. This group may face similar challenges during menopause, but also face their own specific challenges. We refer to the work of Tania Glyde for the specific challenges which non-binary or queer people may face during menopause.

## Declarations

### Author information

ET (he/him) is a medical doctor and PhD student studying gendered experiences during ageing. HR-M (she/her) and MM (she/her) are both medical specialists and clinical researchers in geriatric medicine. PV (she/her) trained as a psychologist and is currently an associate professor in gender studies. All the authors are highly educated, white and able-bodied.
